# A Human *TSC1* Variant Screening Platform in Gabaergic Cortical Interneurons for Genotype to Phenotype Assessments

**DOI:** 10.3389/fnmol.2020.573409

**Published:** 2020-09-18

**Authors:** Dean Wundrach, Luis E. Martinetti, April M. Stafford, Stephanie M. Bilinovich, Kartik Angara, Jeremy W. Prokop, Shane R. Crandall, Daniel Vogt

**Affiliations:** ^1^Department of Pediatrics and Human Development, Michigan State University, Grand Rapids, MI, United States; ^2^Department of Physiology, Michigan State University, East Lansing, MI, United States; ^3^Neuroscience Program, Michigan State University, East Lansing, MI, United States; ^4^Department of Pharmacology & Toxicology, Michigan State University, East Lansing, MI, United States; ^5^Center for Research in Autism, Intellectual and other Neurodevelopmental Disabilities, Michigan State University, East Lansing, MI, United States

**Keywords:** *TSC1*, autism (ASD), cortical interneuron, GABA, variant

## Abstract

The *TSC1* and *TSC2* genes are connected to multiple syndromes from Tuberous Sclerosis Complex (TSC) to autism spectrum disorder (ASD), with uncertainty if genetic variants cause all or subsets of phenotypes based on the location and type of change. For *TSC1*, few have addressed if non-TSC associated genetic variants have direct contributions to changes in neurological genotype-to-phenotype impacts, including elevated rates of ASD and seizures. Dominant variants cause TSC, yet *TSC1* has many heritable variants not dominant for TSC that are poorly understood in neurological function, with some associated with ASD. Herein, we examined how missense variants in *TSC1*, R336W, T360N, T393I, S403L, and H732Y, impacted the development of cortical inhibitory interneurons, cell-types whose molecular, cellular, and physiological properties are altered after the loss of mouse *TSC1*. We found these variants complemented a known phenotype caused by loss of *TSC1*, increased cell size. However, distinct variants, particularly S403L showed deficits in complementing an increase in parvalbumin levels and exhibited smaller amplitude after hyperpolarizations. Overall, these data show that subtle phenotypes can be induced by some *TSC1* missense variants and provide an *in vivo* system to assess *TSC1* variants’ neurological impact better.

## Introduction

The identification of neuropsychiatric genetic variants has increased and proved challenging in autism spectrum disorder (ASD), where several genes are implicated. The discovery of ASD-risk genes has elevated our understanding of ASD biology but also prompts the need for novel assays to continue these advancements (Rosti et al., 2014). While the functional impact of some variants can be easy to predict, i.e., loss of function, frameshift, and nonsense, the effect of missense variants has been challenging to predict and validate.

The integration of more advanced variant support using computational tools to prioritize high impact variants with animal or humanized systems to define the physiological outcomes of the variant can build advanced genotype-to-phenotype insights. An ideal method to validate these variants could be to generate a knock-in model for each, which provides an *in vivo* environment for cells to develop. However, this is costly, time-consuming, and inefficient, making it challenging to study variants *in vivo*. To more efficiently understand the impact of missense variants associated with ASD, we developed and validated an *in vivo* approach that can assess the effect of a variant in GABAergic cortical interneurons (CINs; Vogt et al., 2015a, 2018).

CIN dysfunction is implicated in ASD and altered in both humans diagnosed with ASD and in ASD genetic deletion models (Vogt et al., 2015a, 2018; Hoffman et al., 2016; Hashemi et al., 2017; Jung et al., 2017; Soghomonian et al., 2017; Pla et al., 2018; Elbert et al., 2019; Malik et al., 2019; Angara et al., 2020). CINs are derived from the medial and caudal ganglionic eminences (MGE and CGE), as well as the preoptic area (Wonders and Anderson, 2006; Gelman et al., 2011; Hu et al., 2017b). They tangentially migrate long distances to their cortical destinations, laminate the cortex and express unique molecular markers as they assume their diverse roles, each modulating cortical inhibition in distinct ways (Wonders and Anderson, 2006; Miyoshi et al., 2010; Kessaris et al., 2014). Most CINs are derived from the MGE and are delineated *via* the expression of somatostatin (SST) or parvalbumin (PV). PV expression is commonly affected in ASD and associated animal models (Hashemi et al., 2017; Vogt et al., 2018; Malik et al., 2019), suggesting that PV+ CIN properties may provide a common readout of how ASD missense variants could alter neural development.

Genes underlying syndromes associated with high rates of ASD do impact CIN development, especially PV+ CINs (Vogt et al., 2014, 2015a, 2018; Pla et al., 2018). Many of these genes regulate similar cellular processes, e.g., mammalian target of rapamycin (MTOR) signaling. Particularly, pathogenic variants in *TSC1* that inhibit MTOR activity underlies the Tuberous Sclerosis Complex (TSC). Conditional loss of *TSC1* in mouse CINs leads to ectopic expression of PV and aberrant fast-spiking (FS) properties (Malik et al., 2019). However, nothing is known about how the multitude of missense variants in *TSC1* could impact CIN development and the molecular and physiological properties of PV+ CINs. We developed a platform to test human variants of *TSC1* within CINs by combining cell-specific mouse gene deletion with human allele recovery. *TSC1* variants that are low allele frequency throughout the population (based on gnomAD) were tested to study their impact on the CIN function. These variants have conflicting reports regarding involvement in ASD, as they were reported in early studies of ASD patients (Schaaf et al., 2011; Kelleher et al., 2012), while recent data suggests either no disease association or a more complex multifactorial role in disease, making them great candidates for our assay. Each variant is not connected to the dominant TSC syndrome, allowing for direct segregation of TSC vs. ASD, with a particular interest in recessive ASD linked *TSC1* variants. All variants complemented a classical MTOR phenotype, however, distinct variants failed to complement the elevated PV expression associated with the loss of *TSC1*. As a proof of principle, we probed the physiological properties of one variant, and detected decreased action potential (AP) after hyperpolarizations, suggesting subtle phenotypes that may associate with multifactorial ASD. These data demonstrate a sensitive readout for the *TSC1* variant function *in vivo* and provide a platform to assay more challenging variants.

## Materials and Methods

### Animals

*TSC1^Flox^* (Kwiatkowski et al., 2002) and *Ai14* (Madisen et al., 2010) were previously described. Lines were backcrossed to CD-1 mice for at least five generations before experiments began. For timed matings, noon on the day of the vaginal plug was considered embryonic day 0.5. Experimenters were blind to the genotypes of the mice and littermates were used as controls when possible. Since our previous work did not find a difference in sex phenotypes (Malik et al., 2019), both sexes were used. All mouse procedures were performed following the NIH Guidelines for the Care and Use of Laboratory Animals and were approved by the Michigan State University Institutional Animal Care and Use Committee.

### DNA Vector Generation

The *DlxI12b-BG-hTSC1-IRES-Cre* lentiviral DNA vector was previously described (Malik et al., 2019). To generate the *hTSC1* variants, we designed gene blocks (integrated DNA technologies) that included each mutation and flanking endogenous restriction sites that resided within the human *TSC1* gene. Next, the gene blocks were ligated into the *DlxI12b-BG-hTSC1-IRES-Cre* vector (replacing the WT sequence) and then verified using Sanger sequencing.

### *In vitro* Slice Preparation

Coronal cortical slices (300 μm thick) were prepared (between postnatal ages 34 and 56) using methods previously described (Crandall et al., 2015, 2017). Briefly, mice were deeply anesthetized with isoflurane before decapitation. Brains were then quickly removed and placed in a cold (~4°C) oxygenated slicing solution (95% O_2_, 5% CO_2_) containing (in mM): 3 KCl, 1.25 NaH_2_PO_4_, 10 MgSO_4_, 0.5 CaCl_2_, 26 NaHCO_3_, 10 glucose, and 234 sucrose. Slices were cut using a vibrating tissue microtome (Leica VT1200S) and then transferred into a chamber containing warm (32°C) oxygenated (95% O_2_, 5% CO_2_) artificial cerebrospinal fluid (ACSF) containing (in mM): 126 NaCl, 3 KCl, 1.25 NaH_2_PO_4_, 2 MgSO_4_, 2 CaCl_2_, 26 NaHCO_3_, and 10 glucose. Slices were kept at 32°C for 20 min followed by room temperature for an additional 40 min before recording.

### *In vitro* Electrophysiological Recordings, Data Acquisition, and Analysis

For recordings, individual slices were transferred to a submersion recording chamber and continually perfused (~3 ml/min) with warm (32°C) oxygenated (95% O_2_, 5% CO_2_) ACSF containing (in mM): 126 NaCl, 3 KCl, 1.25 NaH_2_PO_4_, 2 MgSO_4_, 2 CaCl_2_, 26 NaHCO_3_, and 10 glucose. Neurons were visualized using infrared differential interference contrast (IR-DIC) and fluorescence imaging using a Zeiss Axio Examiner.A1 microscope mounted with a video camera (Olympus XM10-IR) and a 40x water-immersion objective. Whole-cell recordings were obtained from tdTomato+ CINs using borosilicate glass pipettes (4–6 MΩ tip resistance) containing a potassium-based internal solution (mM): 130 K-gluconate, 4 KCl, 2 NaCl, 10 HEPES, 0.2 EGTA, 4 ATP-Mg, 0.3 GTP-Tris and 14 phosphocreatine-K (pH 7.25, 290 mOsm). All whole-cell recordings were corrected for a 14-mV liquid junction potential.

Data were recorded and digitized at 20 or 50 kHz using Molecular Devices hardware and software (MultiClamp 700B amplifier, Digidata 1550B4, and pClamp11.1). Signals were low-pass filtered at 10 kHz before digitizing. During the recordings, the pipette capacitances were neutralized and series resistances (typically between 10 and 25 MΩ) were compensated online (100% for current-clamp). Series resistances were continually monitored throughout the recordings.

Analysis of electrophysiological data was performed in Molecular Devices and Microsoft Excel as described (Crandall et al., 2017). Resting membrane potentials (RMP, mV) were measured after the break-in, with no applied current. Input resistance (Rin, MΩ) was measured using Ohm’s law by measuring the voltage response from rest to an injection of a small negative current (5–20 pA). Membrane time constants (τm, ms) were measured from the average response (see Rin above) by fitting a single exponential to the initial falling phase of the response (100–300 ms; omitting the 1st ms). Membrane capacitance (Cin, pF) was calculated by τm/Rin. Rheobase currents (pA) were defined as the minimum positive current (5 pA steps) to elicit an AP from a holding potential of −79 mV. AP properties were measured from the first spike evoked by the rheobase current. AP threshold (mV) was defined as the membrane potential at which its first derivative (*d*V/*d*t) exceeded 10 mV/ms. AP amplitudes (mV) were defined as the voltage difference between the threshold and the peak of the AP. AP half-widths (ms) were measured at the half-height between the threshold and the AP peak. The max rate of rise and decay (mV/ms) was defined as the maximal *d*V/*d*t during the rising phase and falling phase of the same AP, respectively. Fast afterhyperpolarization potentials (fAHPs, mV) were measured as the difference between the AP threshold and the peak negative potential of the AHP immediately following the AP. Post train medium afterhyperpolarization potentials (mAHPs, mV) were measured as the difference between a baseline period (500 ms) before the current injection and the peak negative potential following the 1-s train of APs. Analyses were performed on the first suprathreshold current injection when the initial firing frequency of the cell exceeded 150 Hz, and at least 100 APs were evoked. Membrane potential sags (mV) were measured using a 1 s negative current step that hyperpolarized the neuron from −79 to −99 mV and calculated relative to the steady-state voltage at the end of the step. Frequency-intensity (F/I) relationships were obtained by holding the soma at −79 mV with intracellular current and injecting suprathreshold positive current (50 pA steps, 1 s duration). F/I slopes (Hz/pA) were determined using the initial frequency (reciprocal of the first interspike interval) over the entire F-I plot. Spike frequency adaptation was determined by calculating the adaptation ratio, defined as the steady-state firing frequency (average of the last 5 APs) divided by the initial frequency. Spike height accommodation was defined as the amplitude of the last AP divided by the first AP. Analysis for both spike frequency adaptation and spike height accommodation was performed on the first suprathreshold current injection in which the initial firing frequency exceeded 150 Hz.

### Immuno-fluorescence Labeling and Imaging

Primary neurons and coronal brain sections were washed in PBS containing 0.3% Triton-X100, blocked in the same solution containing 5% BSA, and then incubated in primary antibodies for 1–2 h. They were then washed three times and then incubated with secondary antibodies containing fluorophores for 1 h before three final washes. Primary antibodies included rabbit anti-GABA 1:500 (Sigma A2052), rabbit anti-parvalbumin 1:400 (Swant, PV-27). Alexa-conjugated secondary antibodies (Thermo Fisher) were used to detect primary antibodies. Native tdTomato fluorescence was imaged, and *in vitro* primary, MGE cultures were also labeled for DAPI using NucBlue Fixed Cell ReadyProbes (Thermo Fisher, R37609). A Nikon eclipse Ts2R microscope (Photometrics CoolSnap dyno camera) and Leica DM2000 microscope (DFC3000G camera) captured the primary culture and transplant images, respectively.

### Lentivirus Preparation

*TSC1* lentiviral vectors were co-transfected with *pVSV-g*, *pRSVr*, and *pMDLg-pRRE* plasmids using Lipofectamine^2000^ (Thermo Fisher Scientific) into HEK293T cells, and the media replaced after 4 h, as described (Vogt et al., 2015b). Four days after transfection, the media was collected and filtered to remove debris, then complexed with Lenti-X concentrator (Clontech) according to the manufacturer’s protocol to concentrate lentiviral particles. Lentiviruses were stored at −80°C until use.

### MGE Primary Cultures

We performed MGE primary cultures as described in Angara et al. (2020). Briefly, we cultured MGE cells in DMEM supplemented with 10% FBS and penicillin/streptomycin from time of seeding until 1 day *in vitro*. The cells were transduced with the virus at this stage for 4 h. After 4 h of transduction, we replaced with neurobasal media, supplemented with B27, glucose, glutamax, and penicillin/streptomycin. Cells grew in this media until 7 days *in vitro*, then fixed in 4% paraformaldehyde and assessed *via* immuno-fluorescence.

### MGE Transplantation

Transduced MGE cells were transplanted into neonatal mouse cortices as previously described (Vogt et al., 2015b). Briefly, *Ai14^Flox/+^* E13.5 MGE cells that were either WT, *TSC1^Flox/+^*, or *TSC1^Flox/Flox^* were transduced with *hTSC1-Cre* lentiviruses and then transplanted into WT mouse neonatal cortices at multiple sites. The cells developed *in vivo* for 35 days. Then, cells were identified by native tdTomato expression and were co-labeled for molecular markers *via* immuno-fluorescence.

### Statistics and Cell Assessments

Graphpad Prism 7 and Origin Pro 2019 were used to calculate statistical significance; a *p*-value of < 0.05 was considered significant. For data with parametric measurements, we used a One-Way ANOVA with a Tukey post-test or a Two-sample *t*-test to determine significance. For non-parametric data sets (transplantation experiments where data were normalized), we used a Chi-squared test to determine the significance or a Mann–Whitney U test.

### Western Blotting

Cell pellets were lysed using RIPA buffer. Each sample was diluted to a concentration of 1 mg/mL in a buffer containing protein loading dye. 10 uL of the lysate was loaded into a bis-tris protein gel (4–12% Invitrogen Bolt Bis-Tris mini gel), ran at 150 V for 35 min then transferred to the PVDF membrane using the iBlot2 system. Blots were blocked with 5% milk in TBST for 1 h before incubation with primary antibody (1:1,000 in TBST + 5% BSA) for 1 h at room temperature. Blots were washed four times with TBST and incubated with secondary antibody (1:2,000, HRP-conjugated anti-rabbit, BioRad) for 1 h at room temperature then washed four times with TBST. Signal was detected using SuperSignal West Femto (ThermoScientific) chemiluminescent substrate and imaged on a ChemiDoc system (BioRad). Primary antibodies included rabbit anti-HAMARTIN (Cell Signaling Technologies, 4906) and rabbit anti-GAPDH (Cell Signaling Technologies, 2118).

## Results

### Generation and Validation of Human TSC1 Variants Associated with ASD

**Figure 1:**
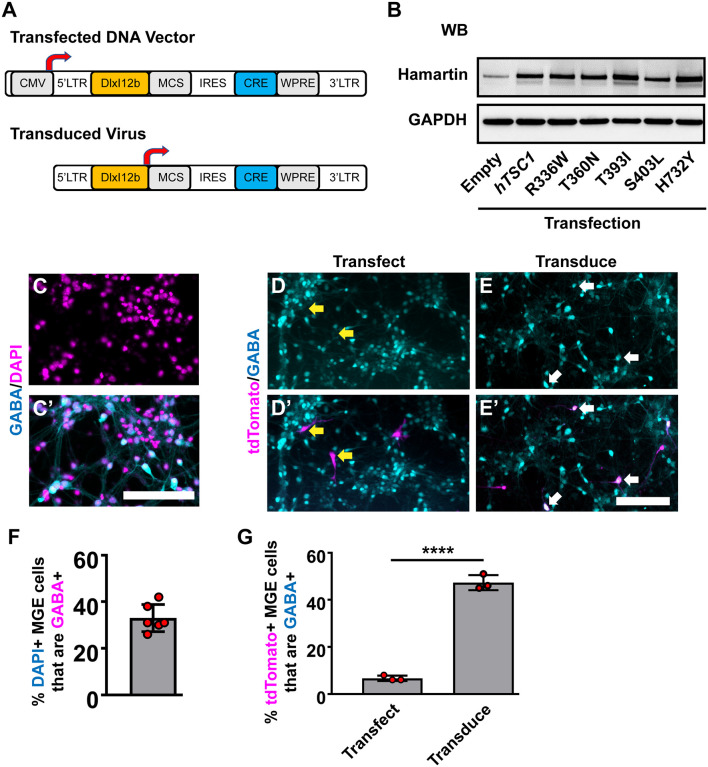
Human *TSC1* variants and expression in medial ganglionic eminences (MGE) primary cultures.** (A)** Schema depicting the lentiviral DNA vector and resulting lentivirus to express human *TSC1* variants and Cre recombinase. **(B)** *TSC1* variants expressed in HEK293T cells show increased levels of expression over endogenous HAMARTIN protein and migration at the correct molecular weight. **(C,C’)** E13.5 MGE primary cultures that were grown for 7 days and co-labeled for GABA and DAPI. E13.5 MGE primary cultures that were either transfected **(D,D’)** or transduced **(E,E’)** with the vector or virus depicted in **(A)** and co-labeled for the Cre-dependent reporter, tdTomato, and GABA. **(F)** Quantification of the proportion of DAPI+ cells that express GABA in MGE cultures. **(G)** Quantification of the proportion of tdTomato+ cells that are GABA+. Yellow arrows denote non-co-labeled cells while white arrows denote co-labeled cells. Data are expressed as the mean ± SEM. Data were collected from three biological replicates (transplants) for all groups. Abbreviations: WB, western blot; KD, kilodalton. Scale bars in **(C’,E’)** = 100 μm. ****p < 0.0001.

We chose five human (h)*TSC1* variants based on their previous association with an ASD diagnosis (Schaaf et al., 2011; Kelleher et al., 2012); R336W, T360N, T393I, S403L, and H732Y, and subcloned them into a lentiviral DNA vector (Figure 1A, top). All five variants are annotated as either conflicting or benign in ClinVar. We first assessed the expression of these variants in our lentiviral vector. While the DNA vector can drive expression from a ubiquitous CMV promoter, the resulting virus uses the *DlxI12b* enhancer, which biases expression to GABAergic neurons (Arguello et al., 2013). Each version expresses either Cre recombinase alone or in combination with WT *hTSC1* or each of the variants. To assess if the variants generated proteins at the correct molecular weight, we transfected HEK293T cells and assessed for the *hTSC1*-encoded protein, Hamartin. Both the WT and variants expressed at elevated levels over endogenous protein and at the correct size, indicating that we could express both WT and variants (Figure 1B).

Next, we utilized MGE primary cultures to test these vectors and viruses *in vitro*. Since cells in the developing MGE are heterogeneous, we asked what proportion of cells were GABAergic in primary E13.5 MGE cultures that had grown *in vitro* for 7 days. 30–40% of the cells were GABAergic (Figures 1C,C′). We either transfected the WT *hTSC1* expression vector or transduced the virus into MGE cultures 24 h after seeding and assessed how many GABAergic cells co-labeled with tdTomato (Cre-expressing) after six additional days. Only ~6% of transfected cells co-labeled for GABA and tdTomato, despite 30–40% of the cultures being GABA+ (Figures 1D,D′,G). However, the *DlxI12b* virus transduced cells had ~50% co-labeled cells. Thus, utilizing the *DlxI12b* enhancer virus was more efficient at biasing towards GABAergic cells but cannot override the strong CMV promoter in the DNA vector. Finally, we transduced the various viruses into the MGE cultures in the same manner but by day 7 *in vitro* did not observe gross differences in cell morphology (data not shown), however, whether phenotypes would emerge at later developmental ages were still unknown.

### *In vivo* Assay to Determine the Impact of hTSC1 Missense Variants

**Figure 2:**
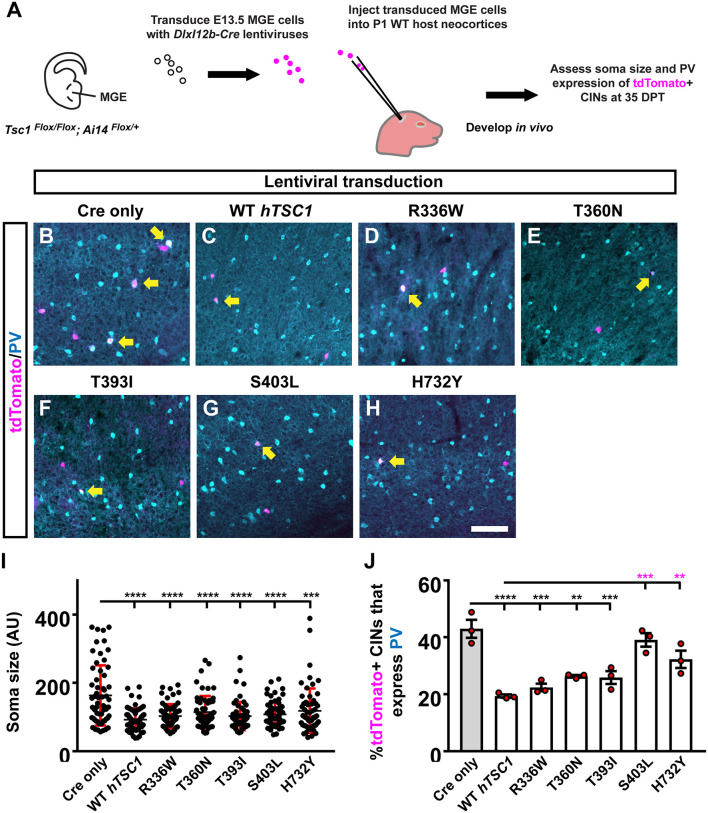
Distinct *TSC1* variants impact PV expression but not soma size. Schema depicting the complementation assay. **(A)** Briefly, *TSC1^Flox/Flox^; Ai14^Flox/Flox^* E13.5 MGE cells were dissociated, transduced with Cre-expressing viruses, transplanted into WT neocortices, and assessed after developing *in vivo* for 35 days. **(B–H)** Example immuno-fluorescent images of transplanted cortical interneurons (CINs) in the neocortex co-labeled for tdTomato and parvalbumin (PV). Arrows point to co-labeled cells. **(I)** Quantification of the soma size for each variant complementation. AU, arbitrary units. **(J)** Quantification of the %tdTomato+ CINs that express PV. Data are expressed as the mean ± SD for soma size and SEM for PV counts, *n* = 3 for all groups. For soma size a total of 75 cells were counted for each group and for PV labeling the number of tdTomato+ CINs assessed were: Cre only 454, WT 152, R336W 205, T360N 156, T393I 295, S403L 148, and H732Y 137. **p < 0.01; ***p < 0.001; ****p < 0.0001. Scale bar in **(H)** = 100 μm.

To understand the impact of *hTSC1* variants in MGE CINs at later ages, we modified an *in vivo* assay (Vogt et al., 2015b) to assess each variant, which allows MGE cells to develop over a long period after genetic manipulation (Figure 2A). We transduced E13.5 MGE cells that were *TSC1^Flox/Flox^*; *Ai14^Flox/+^* with a lentivirus carrying genes for *Cre* and either nothing (Empty), a WT *hTSC1*, or the variants. After transduction, the cells were transplanted into a postnatal day (P)1 WT pup’s cortex to develop *in vivo*. tdTomato+ CINs were assessed 35 days later for soma size and PV expression that are mouse *TSC1* deletion phenotypes (Malik et al., 2019). Consistent with these findings, using a Cre only virus to delete mouse *TSC1* from MGE-lineage CINs resulted in greater soma size and over 40% of CINs expressing PV (Figures 2B,I,J). Expression of WT *hTSC1* complemented these phenotypes (Figures 2C,I,J). All five variants complemented the increase in soma size (vs. Cre, *p* < 0.0001 all variants except H732Y, *p* = 0.0001), and variants resembled WT (Figures 2B–I).

While increased soma size is associated with loss of *TSC1/2* gene function, it often only occurs with the complete ablation of both *TSC1/2* alleles (Tavazoie et al., 2005; Malik et al., 2019), suggesting the variants are functional. Next, we asked whether the expression of PV may be a more sensitive readout to screen the impact of *hTSC1* missense variants as observed in other ASD risk genes (Vogt et al., 2015a, 2018). We assessed the number of tdTomato+ CINs that expressed PV. Two variants failed to complement the increase in PV expression, i.e., S403L and H732Y (Figures 2G,H,J; S403L, *p* = 0.0003, H732Y, *p* = 0.004), suggesting these variants impact CIN molecular properties. While the other variants were not significantly different than WT *hTSC1*, they, like the WT version, were significantly different than Cre-mediated loss of mouse *TSC1* (Figures 2B–F,J; WT, *p* < 0.0001, R336W, *p* = 0.0002, T360N, *p* = 0.009, T393I, *p* = 0.0007). These data demonstrate PV expression as a sensitive *hTSC1*-linked phenotype and specific variants are more likely to affect CIN molecular properties.

**Figure 3:**
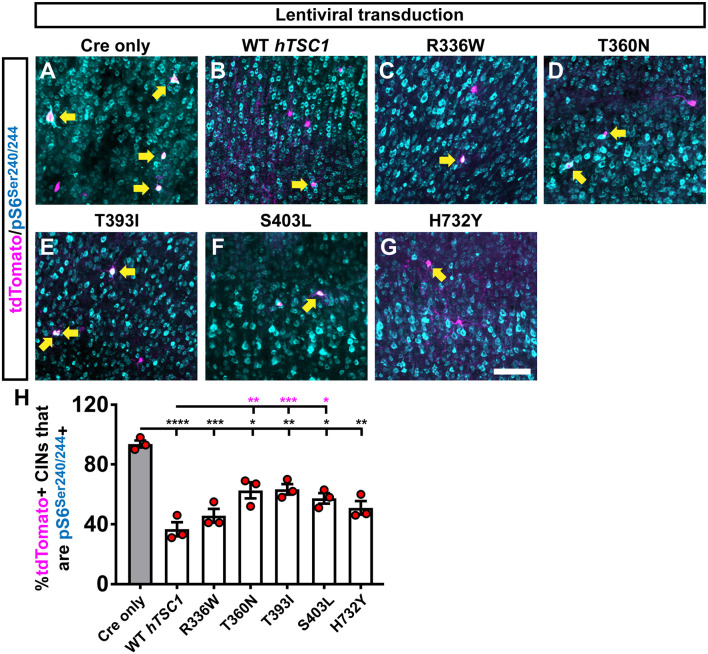
Multiple variants suppress an mammalian target of rapamycin (MTOR) activity target but less efficiently than WT *TSC1*. CINs were assessed 35 days after transduction with the virus and transplantation into WT neocortices of host pups. CINs (tdTomato) that are co-labeled for the MTOR activity marker, phosphorylated (p)S6 at Serines 240 and 244 **(A–G)**; arrows point to co-labeled cells. **(H)** Quantification of the proportion of tdTomato+ CINs that co-label with pS6 for each virus. Data are expressed as the mean ± SEM, *n* = 3 for all groups. The number of tdTomato+ CINs assessed were: Cre only 318, WT 289, R336W 149, T360N 174, T393I 430, S403L 160 and H732Y 164 *p < 0.05; **p < 0.01; ***p < 0.001; ****p < 0.0001. Black bars are *p*-values vs. Cre only; magenta bars are *p*-values vs. WT *hTSC1*. Scale bar in **(G)** = 100 μm.

Finally, we asked if the increased PV expression in the S403L and H732Y variants correlated with elevated MTOR activity by labeling for phosphorylated ribosomal subunit (pS)6. As expected, the deletion of mouse *TSC1* led to greater than 90% of transplanted cells positive for pS6, and expression of human WT *TSC1* brought these levels down to ~40% (Figures 3A,B,H, *p* < 0.0001). All of the *TSC1* variants resulted in significantly lower levels of pS6 compared to Cre only (Figures 3C–H, R336W, p = 0.0004, T360N, *p* = 0.045, T393I, *p* = 0.008, S403L, *p* = 0.02, H732Y, *p* = 0.004). However, three variants were slightly hypofunctional compared to WT *TSC1* (Figure 3H, T360N, *p* = 0.003, T393I, *p* = 0.0004, S403L, *p* = 0.01). While the S403L variant was one of the variants that deviated, T360N and T393I had the highest deviation from WT pS6 levels, suggesting that MTOR activity through elevated pS6 may not be the primary mechanism underlying the increase in PV expression.

### Afterhyperpolariztions Are Abnormal in S403L CINs with FS Properties

**Figure 4:**
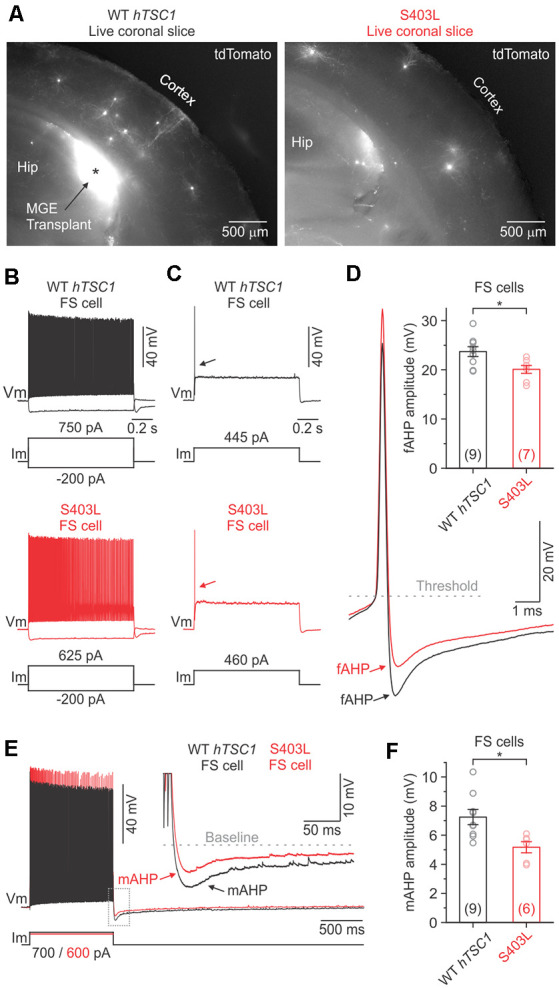
Fast and medium afterhyperpolarization are reduced in S403L CINs with fast-spiking (FS) properties.** (A)** Low-magnification fluorescence images taken of live brain sections (300 μm) showing tdTomato-expressing MGE cells transduced with either the WT *TSC1* or S403L missense variant after developing *in vivo*. **(B)** Voltage responses of a WT *TSC1* and S403L CIN to intracellular current steps (1 s). Both cells were classified as FS using a non-linear classifier based on key electrophysiological properties (see “Results” section). **(C)** Responses to a threshold current injection (rheobase) for the same cells shown in **(B)**. **(D)** Left, superimposed are the indicated single APs from (**C**: arrows) at increased magnification. Note the smaller fast afterhyperpolarization potential (fAHP) amplitude immediately following the action potential (AP) for the S403L cell. fAHPs were measured as the difference between the AP threshold and the peak negative potential. Right, quantification of the fAHP amplitude for each variant (fAHP WT *TSC1*: 23.7 ± 1.0 ms, *n* = 9 cells from four mice; fAHP S403L: 20.1 ± 0.8 ms, *n* = 7 cells from four mice; *p* < 0.02, two-sample t-test, two-tailed). **(E)** Spike trains evoked by a positive injected current. Traces are from the same cells shown in **(B)**. Inset, showing the post-train mAHP at increased magnification. Note the smaller mAHP amplitude immediately following the spike train for the S403 > L cell. **(F)** Quantification of the post-train mAHP amplitude for each variant (mAHP WT *TSC1*: 7.2 ± 0.5 ms, *n* = 9 cells from four mice; mAHP S403L: 5.2 ± 0.4 ms, *n* = 6 cells from four mice; *p* < 0.02, two-sample t-test, two-tailed). Data are expressed as the mean ± SEM. *p < 0.05.

To further explore the possibilities of this assay, we chose to focus on the S403L variant, which uniquely resulted in both elevated PV and pS6 levels. PV is an EF-hand calcium-binding protein found in distinct classes of neurons and is likely to play a role in the behavior of these cells (Celio, 1986). To investigate the functional effects of the S403L variant, we made whole-cell recordings of transduced MGE CINs in the somatosensory cortex of similarly aged littermate mice transplanted with cells expressing the S403L variant or the WT *hTSC1*. Transduced cells were identified by their tdTomato expression (Figure 4A). As a population, the passive and active membrane properties of S403L CINs were not significantly different from WT *hTSC1* cells (Supplementary Table S1).

The S403L variant may lead to more CINs with FS properties, like *TSC1* loss of function (Malik et al., 2019). To verify cell identity objectively, we used a simple non-linear classifier based on three electrophysiological properties (Hu et al., 2013), i.e., AP half-width (APHW), afterhyperpolarization amplitude (AHP), and spike frequency adaptation ratio (AR), distinct properties typical of FS CINs. Cells were classified as FS if at least two of the following conditions were true: APHW < 0.26 ms, AHP > 16.5 mV, and AR > 0.56. Cells not meeting these conditions were classified as non-FS cells (i.e., putative SST+ CINs). When we applied the classifier to our population of S403L CINs, we found that 56.3 ± 9.2% of the cells were classified as FS (*n* = 4 mice). Data from WT *hTSC1* CINs, however, yielded a similar percentage of FS cells (45.0 ± 12.6%; *n* = 5 mice; *p* = 0.52).

The intrinsic membrane properties of S403L FS CINs were similar to WT *hTSC1* FS cells in their resting potential, input resistance, membrane time constant, and capacitance (Supplementary Table S2). The latter consistent with similar soma sizes (Figure 2I). Analysis of APs confirmed that cells classified as FS had electrophysiological properties typical of FS CINs, i.e., high-frequency discharge patterns with little spike-frequency adaptation (average adaptation ratios: 0.61 for WT *hTSC1*, 0.75 for S403L; Figure 4L). Only one S403L cell was not capable of sustained AP discharge throughout a 1-s suprathreshold current injection. Interestingly, analysis of single APs evoked by a threshold current injection revealed a smaller fast afterhyperpolarization (fAHP) immediately following the AP in S403L than WT *hTSC1* cells (Figures 4C,D). Further, we found that the peak medium AHP (mAHP) following a 1-s spike train was smaller for S403L than WT *hTSC1* cells (Figures 4E,F). We found no differences in the functional properties between S403L and WT *hTSC1* cells classified as non-FS (Supplementary Table S2). Overall, the physiological data indicate that the S403L missense variant results in both a reduced fAHP and mAHPs in CINs classified as FS.

## Discussion

We modified an *in vivo* assay (Vogt et al., 2014, 2015a, 2018; Hu et al., 2017a; Pla et al., 2018) to understand the impact of human variants in CINs. By examining a gene underlying a syndrome with high rates of ASD, i.e., *hTSC1*, we discovered that missense variants could complement a common phenotype used to assess *hTSC1* dysfunction, i.e., increased cell size, suggesting that this measurement is a poor rheostat to assess what impacts these variants cause. However, some variants did not complement altered PV expression, suggesting that this measurement may be more sensitive to assess variants. Finally, while *TSC1* has several conserved protein domains, less is known about the protein’s function. While fewer missense variants are found in *TSC1* compared to *TSC2* their assessment may elucidate the role of *TSC1*’s unstudied domains.

Why two of the variants were deficient at complementing the PV expression phenotypes is still unknown. They are found in mid to carboxy-terminal regions of Hamartin, which could imply distinct protein domains or unique functions of these regions. Currently, we do not understand why these variants would alter CIN properties given what has been reported for each the variants (Schaaf et al., 2011; Kelleher et al., 2012; Bahl et al., 2013); each is found in an individual diagnosed with ASD but not TSC and no other comorbid symptoms or studies have been reported. One hypothesis is that the S403L variant is a potential serine phosphorylation site that has not been examined, which may reveal novel MTOR pathway regulation as we proceed. Also, cell-type-specific enhancers are great at enriching for cell groups of interest but are still imperfect (Figure 1), especially when expressing Cre, which can be active at low levels. We believe this is still a great approach for our *in vivo* assay, as the cells being analyzed must migrate out of the transplant site, which is primarily GABAergic CINs and other MGE cells with off-target viral expression should remain in the injection site. However, we caution using *Dlx* enhancers to express Cre in direct injection assays, i.e., injecting AAVs into a discrete region as many recombined cells may not be GABAergic.

The S403L variant resulted in a subtle reduction of the fAHP following a single AP and the mAHP following a train of APs in CINs with FS properties, with no apparent changes to passive membrane properties. This result differs from the deletion of *TSC1* in mice, which have several alterations in both passive and active membrane properties (Normand et al., 2013; Kosillo et al., 2019; Malik et al., 2019). AHPs play an essential role in shaping neuronal firing properties (Hille, 2001). Although we did not see any gross differences in the AP discharge properties of S403L compared to WT *hTSC1* FS CINs, there could be other compensatory mechanisms at play. The S403L missense variant also increased the number of PV-expressing CINs and is potentially relevant to our physiological observation due to PV’s calcium-binding abilities. If MGE-derived CINs overexpress PV, it could modulate the intracellular calcium dynamics that occur during APs. Indeed, different calcium-activated potassium channels are responsible for the various types of AHPs in mammalian neurons (Hille, 2001; Faber and Sah, 2003). Notably, we found that a marker of elevated MTOR activity, pS6, does not correlate with the increase in PV expression (Figures 2, 3), however, our previous work suggested that the increase in PV expression may be a separate event (Malik et al., 2019) that needs to be further investigated.

We demonstrate that unique variants in *hTSC1* can impact CIN properties. Sensitive changes in CINs can occur *via* single amino acid changes in *hTSC1*, while common phenotypes, i.e., increased cell size, were not observed. This is important as PV expression is responsive to MTOR activity (Malik et al., 2019). Thus, PV expression and CIN function may be sensitive readouts in TSC and a means to understand the impact of the growing number of ASD variants. These data support the idea that mild changes in the function of critical cellular signaling events may be a factor influencing the development and maturation of these unique neurons potentially in ASD and independent of the dominant genetic linked TSC.

## Data Availability Statement

The raw data supporting the conclusions of this article will be made available by the authors, without undue reservation, to any qualified researcher.

## Ethics Statement

The animal study was reviewed and approved by the Michigan State University Institutional Animal Care and Use Committee.

## Author Contributions

DW performed primary cultures and analyses. SB performed western blots. KA performed blinded cell counts. LM performed physiology experiments. AS and DV performed MGE transplants. DW, LM, SC, JP, and DV planned out experiments and wrote the article. All authors contributed to the article and approved the submitted version.

## Conflict of Interest

The authors declare that the research was conducted in the absence of any commercial or financial relationships that could be construed as a potential conflict of interest.
